# Bispicolyamine-Based Supramolecular Polymeric Gels Induced by Distinct Different Driving Forces with and Without Zn^2+^

**DOI:** 10.3390/ijms21134617

**Published:** 2020-06-29

**Authors:** Jaehyeon Park, Ka Young Kim, Seok Gyu Kang, Shim Sung Lee, Ji Ha Lee, Jong Hwa Jung

**Affiliations:** 1 Department of Chemistry and Research Institute of Natural Sciences, Gyeongsang National University, Jinju 52828, Korea; parkjae@gnu.ac.kr (J.P.); rk5321@gnu.ac.kr (K.Y.K.); dsa61milk@naver.com (S.G.K.); sslee@gnu.ac.kr (S.S.L.); 2 Chemical Engineering Program, Graduate School of Advanced Science and Engineering, Hiroshima University, 1-4-1 Kagamiyama, Higashi-Hiroshima, Hiroshima 739-8527, Japan

**Keywords:** bispicolylamine, complexes, gels, zinc, supramolecular gels

## Abstract

Metal-coordination polymeric gels are interesting areas as organic/inorganic hybrid supramolecular materials. The bispicolylamine (BPA) based gelator (**1**) showed excellent gelation with typical fibrillar morphology in acetonitrile. Upon complexing **1** with Zn^2+^, complexes ([**1** + Zn + ACN]^2+^ and [**1** + zinc trifluoromethanesulfonate (ZnOTf)]^+^) with four coordination numbers were formed, which determine the gel structure significantly. A gel-sol transition was induced, driven by the ratio of the two metal complexes produced. Through nuclear magnetic resonance analysis, the driving forces in the gel formation (i.e., hydrogen-bonding and π–π stacking) were observed in detail. In the absence and the presence of Zn^2+^, the intermolecular hydrogen-bonds and π–π stacking were the primary driving forces in the gel formation, respectively. In addition, the supramolecular gels exhibited a monolayer lamellar structure irrespective of Zn^2+^. Conclusively, the gels’ elasticity and viscosity reduced in the presence of Zn^2+^.

## 1. Introduction

Gelating compounds are often used to aid the self-assemblage of nanostructures with predetermined physical properties [1,2,3,4]. To achieve that function, the required driving forces are weak, non-covalent interactions (such as intermolecular hydrogen-bonding, π–π stacking, electrostatic interaction, charge-transfer, metal–ligand coordination, etc.) [5,6,7]. Usually, the weak interactions make supramolecular gels susceptible to various external stimuli. However, the aforementioned bonding systems are easily modulated to form a variety of self-assembled structures and functions at the molecular level. Therefore, molecular design for various self-assembled structures is an emerging and important field [8,9,10,11,12,13]. Yet, previous research suggests that gelators are not discovered by chance. Rather, they are intentionally synthesized based on adequate understanding of their supramolecular interactions.

Metal triggered-supramolecular gels based on the metal–ligand complex have also attracted much attention as a significant class of organic/inorganic hybrid supramolecular materials [14,15,16,17,18,19,20,21]. Due to the combination of metal complexes in metal triggered-supramolecular gels, these metal-containing systems exhibit redox, optical, electrochromic, catalytic, and magnetic properties [22,23,24,25,26]. In particular, chiral metal triggered-supramolecular gels are attractive because they are applicable to chiral recognition and sensing, and asymmetric catalysis [27,28]. For example, the Vivian Wing-Wah Yam [29,30,31] and Chi-Ming Che [16,32] groups reported supramolecular polymerization based on luminescent platinum(II) complexes that exhibit a square planar structure, which are made of covalent bonds between the ligands and platinum(II). Their research focused on morphology transitions and length control of supramolecular fibers with living polymerization. Our group has also investigated on morphology transformation and enhancement of strength of supramolecular gel with metal ions, such as Pt^2+^ and Zn^2+^ [33,34].

Bispicolylamine (BPA)-based ligands are popular for having metal-binding sites of three nitrogen donors that afford good selectivity, especially for Zn^2+^ [35]. It is also well-known that tridentate ligands are capable of donating three electron pairs to a cationic metal center [36]. This is because of the conformational flexibility of BPA. Accordingly, BPA ligands have been used as such, but more widely as linked ligand units in larger host structures [37,38,39]. There are examples on BPA-functionalized metal–anion fluorescence probe linked host molecules [39,40,41,42]. In another example, the bimetallic double BPA chelates can act to the molecular recognition of anionic species, such as phosphate [43,44], pyrophosphate [45,46], or phospholipid [47] anions, as well as histidine residues, to some extent. Thus, the BPA-based ligand has a meaning from a biological point of view, as well as a metal ligand complex.

**Figure 1:**
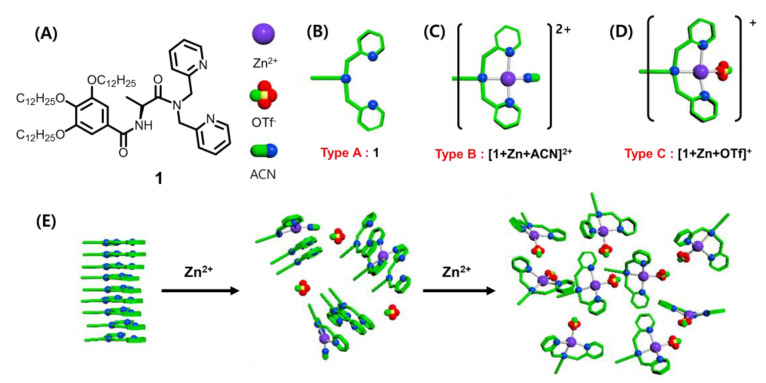
(**A**) Chemical structure of gelator **1**. Proposed structure of (**B**) free ligand, (**C**) [1+Zn^2+^+ACN]^2+^, and (**D**) [**1**+Zn^2+^OTf^-^]^+^; A type without Zn^2+^, B type at 0.1–0.4 equiv. of Zn^2+^, B and C types at 0.5–0.7 equiv. of Zn^2+^ and C type at above 0.8–1.0 equiv. of Zn^2+^ (**E**) The illustration of the expected structure conformation at different amount of Zn^2+^.

In the current work, we produced a BPA-based gelator by introducing *L*-alanine and long alkyl chain in predesigned solvent conditions (Figure 1A). The idea was to induce metal-binding to modify the gel behavior. Thus far, the available research on coordination geometries between BPA and Zn^2+^ have reported mainly tetrahedral, square pyramidal, trigonal bipyramidal, and octahedral structures [48,49,50,51,52]. Having those varieties indicates that the coordination number of the complex and the ligand-to-metal ratio could significantly influence the self-assemblage orientation of the molecules. Furthermore, sol-gel transitions could be controlled by altering some external stimuli, such as pH, temperature, or ultrasound [53,54,55,56]. Similar conditions are required for the metal-binding in a network structure that forms the gel. However, our research investigated the influence of the structure or relative amount of the complexes on the gel–sol transition. Here we describe the effect of complex structures (Figure 1B–D) with four coordination number formed at four stages (equiv.) in the supramolecular gel. Finally, through ^1^H nuclear magnetic resonance (NMR) analysis, the driving forces in supramolecular gels were verified.

This study observes the formation and characterization of supramolecular gel depending on the structure of the complex and the presence ratio of the complexes. There are many single crystal studies on metal coordination structures, but few studies on gels in which these metal coordination structures form building blocks. We have been studying these metal coordination gels for a long time. In here, (**1**) a gelator capable of interacting with supramolecular interactions such bispicolylamine moiuey for metal binding, amide groups for hydrogen bond and aromatic group for pi-pi bond was synthesized. (**2**) The gel–sol transition and the change of network structure were investigated while finely controlling the amount of metal. It is thought that the basic research on the structure of supramolecules, which can be controlled in detail based on the supramolecular interaction, is a step closer to the development of new materials.

## 2. Results and Discussion

**Figure 2:**
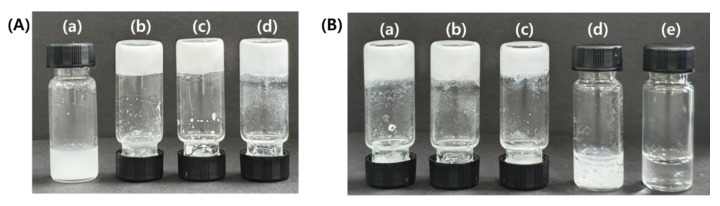
(**A**) Photographs of the gels prepared from gelator **1** (a) 2 mg, (b) 4 mg (c) 6 mg (d) 8 mg in ACN (0.2 mL). (**B**) 8 mg of gelator **1** and (a) 0.1 equiv., (b) 0.3 equiv., (c) 0.5 equiv., (d) 0.7 equiv., (e) 0.9 equiv. of Zn^2+^ in ACN (0.2 mL).

L-alanine-based gelator (**1**) (Figure 1A), which can bind Zn^2+^, was synthesized in four steps (Scheme S1). The gelation tests of **1** were performed under various solvent as shown in Table S1. Gelator **1** could gelate acetonitrile, methanol, and hexane solvents (Table S1). We observed that the critical concentration for the gel formation was 21.6 mM (Figure 2A). In addition, the gelation ability of **1** was evaluated at varying concentrations (≤1.0 equiv.) of Zn(OTf)_2_. The gel formed at <0.5 equiv. of Zn(OTf)_2_. However, at >0.5 equiv., the gel structure broke apart, dissolving in the solvent (Figure 2B). Therefore, we inferred that the complexing of **1** with Zn^2+^ would be critical in the gel formation.

**Figure 3:**
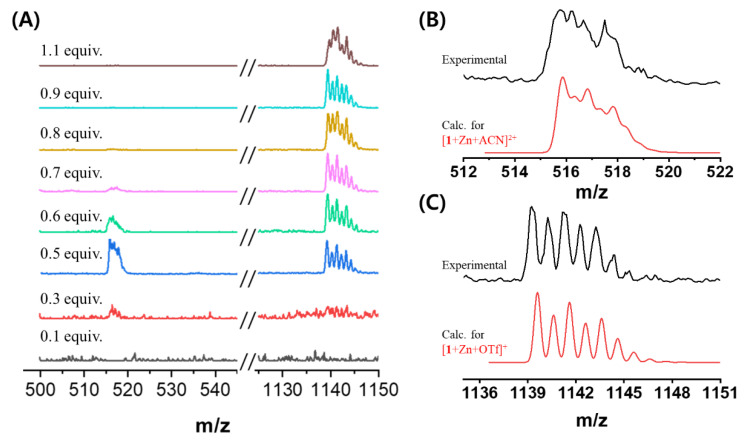
(**A**) Electrospray ionization (ESI)-mass spectra of **1** (0.1 mM) in the various equiv. of Zn^2+^ in ACN. Experimental and calculated data of (**B**) [**1**+Zn+ACN]^2+^ and (**C**) [**1**+Zn+OTf]^+^.

To verify the effect of the complex structure in the gel formation, the stoichiometry ratio between Zn^2+^ and gelator was investigated by electrospray ionization-mass spectroscopy (ESI-MS) (Figure 3 and Table S2). At < 0.5 equiv., the main peak was obtained at *m*/*z* = 515.8385, which corresponds to [**1** + Zn + ACN]^2+^. Besides, the MS data of the experimental isotope for the complex structure matched well with that of the theoretical isotope (Figure 3B,C). We observed that the gelator formed a 1:1 complex with four coordination number. In contrast, at 0.6–0.8 equiv., both [**1** + Zn + ACN]^2+^ (Figure 1B) and [**1** + ZnOTf]^+^ (Figure 1C) coexisted at *m*/*z* = 515.8385 and 1139.6030, respectively. Thus, mixed complex structures at 0.6–0.8 equiv. of Zn^2+^ induced gel collapse. Furthermore, when Zn^2+^ ion was present at > 0.9 equiv., the anion (OTf) coordinated to Zn^2+^ instead of the acetonitrile. Table S2 summarized the ESI-MS results for each equivalent. From the table, we noticed that the 1:1 ratio between **1** and Zn^2+^ remained unchanged, regardless of the amount of Zn^2+^ (Figure 1C,D). It means the amount of Zn^2+^ did not affect the complex ratio. In the condition of the Zn^2+^ equivalent from 0.3 to 1.1, the complex was existed to 1:1 ratio. However, one acetonitrile molecule in the complex was converted into an OTf. When acetonitrile was replaced with an anion, it induced a geometrical change, which affected the gel formation significantly. Therefore, using large quantities of [**1** + ZnOTf]^+^ complex may distort the gel formation.

To investigate changes in the driving force for the gel formation with Zn^2+^, we measured the NMR at varied Zn(OTf)_2_ equivalence (Figure S1). In the absence of Zn^2+^, the ^1^H NMR spectrum showed sharp peaks, whereas, the peaks were broadened in the presence of Zn^2+^. The latter occurred due to two reasons: first, when 0.5 equiv. of Zn^2+^ was added, one type of complex formed via intermolecular interaction between **1** and Zn^2+^, leading to a supramolecular structure. Secondly, the co-existence of two complexes ([**1** + Zn + ACN]^2+^ and [**1** + ZnOTf]^+^) as shown in Figure 3. However, the solution showed a sharp peak again over 0.9 equiv. of Zn^2+^, which was attributed to the conversion of [**1** + Zn + ACN]^2+^ to [**1** + ZnOTf]^+^. Thus, we affirm that the presence of [**1** + ZnOTf]^+^ induced the gel collapse.

**Figure 4:**
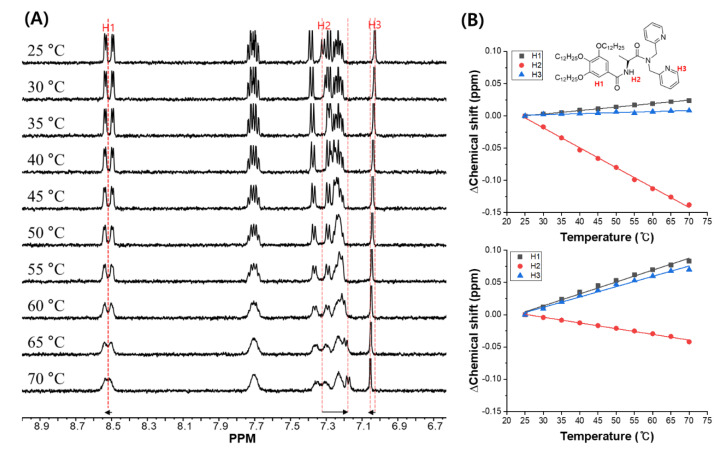
(**A**) Temperature-dependent ^1^H NMR spectra of **1** (2 mM) in ACN-d_3_. (**B**) Plot for chemical shift of H1, H2 and H3 without (upper) and with (below) Zn^2+^ (0.5 equiv.) from 25 to 70 °C.

Additionally, temperature-dependent proton NMR spectra were observed from 25 °C to 70 °C (Figure 4A). In the absence of Zn^2+^, the aromatic protons gradually shifted to low-field by increasing the temperature, which indicated the dissociation of π–π stacking among the aromatic groups. On the contrary, the NH proton shifted to high-field because of the dissociation of the intermolecular hydrogen-bonding. These findings are evidence that **1** formed intermolecular hydrogen-bonding during the gel formation. Interestingly, the degree of chemical shift of NH proton was larger than those of the aromatic protons, which suggests that the intermolecular hydrogen-bonding was much stronger than the π–π stacking.

In the presence of 0.5 equiv. of Zn^2+^, the chemical shift of NH proton was ca. 3.4 fold smaller than that without Zn^2+^ (Figure 4B and Figure S2). This means that the hydrogen bond of NH was weakly existed. The relative chemical shift of the aromatic protons was larger than those in the absence of Zn^2+^, indicating that π–π stacking was the driving force in gel formation in the presence of Zn^2+^. In contrast, the chemical shifts of the aromatic and the NH protons were smaller in the presence of 0.5 equiv. of Zn^2+^, suggesting that the intermolecular interaction during self-assembly of **1** was weakened by Zn^2+^ by formation of complexes (Figure 1C,D). Particularly, we observed a significant difference in the driving force of the gel formation with or without Zn^2+^. The intermolecular hydrogen-bonding interaction between amide and amide groups was the main driving force in the absence of Zn^2+^ whereas, in the presence of Zn^2+^, it was the π–π stacking between aromatic and aromatic groups. In specific, at 1 equiv., the chemical shift of aromatic and NH protons was less than at 0.5 equiv., forming monomeric species due to the weak intermolecular interactions (such as hydrogen-bonding and π–π stacking).

To further examine the intermolecular interaction within the complex, the temperature-dependent UV–vis spectra were measured. As shown in Figure S3, the absorbance of gel **1** increased gradually with or without Zn^2+^, indicating that **1** formed the gel by π–π stacking between aromatic and aromatic groups. Furthermore, the change in absorbance of **1** in the absence of Zn^2+^ was larger than in its absence. Thus, the strength of π–π stacking was stronger in the absence of Zn^2+^.

An additional molecular analysis via Fourier-transform infra-red (FT-IR) spectroscopy confirmed the presence of hydrogen-bonding interaction in **1**, irrespective of Zn(OTf)_2_ (Figure S4). The gel **1** without Zn^2+^ showed the amide NH band at 3244 cm^−1^ and C=O band at 1679 cm^−1^. Whereas, with Zn^2+^ (1.0 equiv.), the wavenumbers shifted to 3325 and 1704 cm^−1^, respectively. These observations implied that the intermolecular hydrogen-bonding of gel **1** was much stronger in the absence of Zn^2+^.

**Figure 5:**
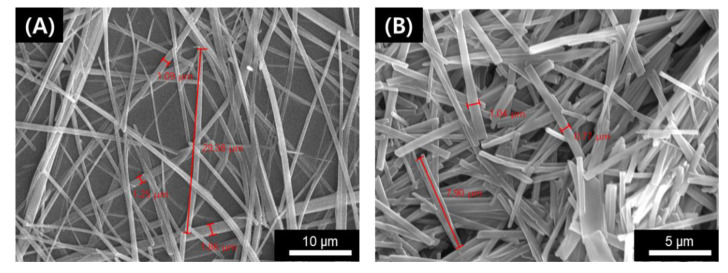
SEM images of xerogels of **1** (**A**) without and (**B**) with Zn^2+^ in ACN.

Further, the X-ray diffraction patterns for gel **1** were measured for a deeper understanding of its molecular assembly after gelation (Figure S5). The small-angle diffraction patterns for gel **1** showed reflection peaks at 2θ = 2.65, 5.39, and 10.60. The ratios of the diffraction peaks were 1, 1/2, and 1/4, which strongly supports lamellae formation. From the diffraction pattern, the observed *d*-spacing (3.3 nm, the length of the fully-extended molecule of **1**) indicated that **1** without Zn^2+^ formed a monolayer lamellar structure. The reflection peak that corresponds to 3.6 Å was also obtained at 2θ = 24.6, indicative of existence of π–π stacking in the gel formation. In contrast, the d-spacing in the presence of Zn^2+^ was 4.0 nm. Again, such difference in *d*-spacing informed that the gel also formed a lamellar, albeit loose, structure with monolayer in the presence of Zn^2+^. Moreover, we observed the morphology of gel **1** without and with Zn^2+^ by scanning electron microscopy. The gel **1** without Zn^2+^ showed a thin and straight fiber structure with 0.5–2 μm of width and ca. 7–10 μm of length (Figure 5A). On the contrary, by adding Zn^2+^ (0.5 equiv.), the length of the fibers shortened due to the collapse of the gel network (Figure 5B).

Finally, we noticed the gel broke when Zn^2+^ was added excessively. To confirm the elasticity (G’) and viscosity (G”) of gel, rheological properties were measured and compared based on the amount of Zn^2+^ added (Figure S6). As expected, with an increase in the amount of Zn^2+^, the G’ and G” of gel decreased, meaning the elasticity and viscosity of gel weakened. However, the reverse points of strain (%) from G’ > G” (i.e., solid behavior) to G” > G’ (i.e., liquid behavior) were 10% to 200%. Such change was attributed to the enhancement of elasticity with the formation of a complex between **1** and Zn^2+^. Besides, we observed a phase change of the gel as heat was applied (Figure S7). There was a noticeable but negligible difference in the time required to melt the gel with or without Zn^2+^. Whereas, the temperatures at which the gel melted completely into a clear solution were similar.

## 3. Materials and Methods

### 3.1. Reagents and Instruments

Precursor **2** was synthesized according to our previously reported procedure [57,58]. Unless otherwise noted, chemical reagents and solvents were purchased from commercial suppliers (Tokyo Chemical Industry (TCI), Sigma Aldrich) and used without further purification. Using a Bruker (ARX 300) and Bruker (DRX-500), the ^1^H and ^13^C NMR spectra of the samples were obtained. The optical absorption spectra of the samples were obtained at 298 K using a UV-vis spectrophotometer (Thermo Evolution 600). A Thermo FT-IR Nicolet iS 10 was used to measure the FT-IR spectra in ATR, in the range of 400–4000 cm^−1^. Mass spectroscopy sample were analyzed on a Thermo Scientific LCQ Fleet mass spectrometer. Powder XRD patterns were measured on a Bruker AXS D8 Advance A25.

### 3.2. Synthesis of N-[3,4,5-Tris(dodecyloxy)]benzoyl L-Alanine Methyl Ester (3)

To a suspension of c N-[3,4,5-Tris(dodecyloxy)]benzoic acid (0.5 g, 0.741 mmoL) in toluene (20 mL) was added SOCl_2_ (0.548 mL, 7.41 mmoL). The reaction mixture was refluxed for 4 h and was cooled to room temperature. The solvent and unreacted SOCl_2_ were removed to give compound. Freshly prepared acid chloride was dissolved in chloroform (10 mL), and L-alanine methyl ester hydrochloride (0.124 g, 0.889 mmoL) was added. The resulting mixture was cooled to 0 °C, and then a solution of triethylamine (0.37 g, 3.70 mmol) in DCM (5 mL) was added dropwise while the temperature was kept below 0 °C. The reaction mixture was stirred at room temperature overnight. Afterwards, it was diluted with DCM (150 mL), and the solution washed three times with distilled water (100 mL), and dried over anhydrous Na_2_SO_4_. The solvent was then removed in vacuo. After the crude product was recrystallized from DCM/ACN and dried to give **3** as white solid in 91% yield (0.51 g). FT-IR (ATR): *v* = 3359, 2917, 2849, 1741, 1575, 1542, 1500 1456, 1425, 1391, 1351, 1317, 1292, 1246, 1224, 1199, 1155, 1115, 996, 872, 833, 770, 720, 615, 573, 551; ^1^H NMR (500 MHz, Chloroform-*d*) δ 7.01 (s, 2H), 6.62 (d, *J* = 7.3 Hz, 1H), 4.80 (t, *J* = 7.2 Hz, 1H), 4.02 (dt, *J* = 11.7, 6.6 Hz, 6H), 3.82 (s, 3H), 1.88–1.73 (m, 6H), 1.54 (d, *J* = 7.1 Hz, 3H), 1.52–1.45 (m, 6H), 1.41–1.21 (m, 48H), 0.91 (t, *J* = 6.9 Hz, 9H).(Figure S8); ^13^C NMR (125 MHz, CDCl_3_) δ 173.83, 166.71, 153.14, 141.47, 128.83, 105.84, 73.52, 69.42, 52.59, 48.55, 31.96, 31.94, 30.33, 29.77, 29.75, 29.72, 29.68, 29.65, 29.62, 29.60, 29.42, 29.38, 26.09, 22.71, 18.69, 14.13 (Figure S9); ESI-MS: calculated for C_47_H_85_NO_6_ [M + H]^+^ 760.64 found 760.33; anal. calculated for C_47_H_85_NO_6_: C, 74.26; H, 11.27; N, 1.84. Found: C, 74.21; H, 11.12; N, 1.81.

### 3.3. Synthesis of N-[3,4,5-Tris(dodecyloxy)]benzoyl L-Alanine (2)

N-[3,4,5-Tris(dodecyloxy)]benzoyl *L*-alanine methyl ester (**3**) (1 g, 1.31 mmoL) and NaOH (0.315 g 7.89 mmol) were dissolved in a mixture of ethanol (30 mL). The solution was heated to reflux for 12 h and then cooled to room temperature. Afterwards, it was acidified with concentrated hydrochloric acid (37%) to pH 2. The resulting precipitate was filtered off and dried in vacuo. After recrystallization from DCM/ACN and dried to give **2** as white solid in 88% yield (0.90 g). FT-IR (ATR): 3360, 2917, 2849, 1746, 1575, 1542, 1500, 1456, 1425, 1391, 1351, 1317, 1292, 1246, 1224, 1199, 1155, 1115, 998, 872, 832, 770, 720, 614, 573, 551. ^1^H NMR (500 MHz, Chloroform-*d*) δ 7.01 (d, *J* = 2.6 Hz, 2H), 6.65 (dd, *J* = 7.0, 2.2 Hz, 1H), 4.87–4.72 (m, 1H), 4.02 (q, *J* = 6.3 Hz, 6H), 1.92–1.72 (m, 6H), 1.60 (dd, *J* = 7.2, 4.1 Hz, 3H), 1.55–1.43 (m, 6H), 1.44–1.23 (m, 48H), 0.90 (t, *J* = 6.8 Hz, 9H) (Figure S10); ^13^C NMR (125 MHz, CDCl_3_) δ 176.30, 153.20, 141.78, 128.12, 105.94, 73.57, 69.47, 31.96, 31.94, 30.33, 29.77, 29.75, 29.72, 29.71, 29.68, 29.66, 29.60, 29.43, 29.41, 29.38, 26.11, 26.09, 22.71, 18.05, 14.13 (Figure S11); ESI-MS: calculated for C_46_H_82_NO_6_ [M - Na]^-^ 744.61 found 744.31; anal. calculated for C_46_H_82_NO_6_: C, 74.05; H, 11.21; N, 1.88. Found: C, 73.91; H,11.31; N, 1.82.

### 3.4. Synthesis of Gelator 1

N-[3,4,5-Tris(dodecyloxy)]benzoyl *L*-Alanine (**2**) (0.2 g, 0.268 mmol) and TBTU (0.086g, 0.268 mmol) were suspended in DCM (10 mL) and DIPEA (0.28 mL, 1.61 mmol) was added. After stirring the reaction mixture for 10 min, the Dipicolylamine (0.053 mg, 0.268 mmol) was added. After stirring overnight at room temperature, the mixture was washed with water (2 times) and the organic phase was dried with Na_2_SO_4_. The organic solvent was removed, and the crude product was purified via column chromatography (silica gel using EA). After recrystallization from DCM/ACN and dried to give **1** as white solid in 80% yield (1.98 g). FT-IR (ATR): 3230, 2916, 2849, 1679, 1648, 1624, 1591, 1578, 1544, 1498, 1467, 1435, 1422, 1407, 1390, 1336, 1305, 1245, 1229, 1211, 1168, 1145, 1107, 1047, 1033, 1014, 993, 865, 767, 749, 720, 675, 636, 613, 457. ^1^H NMR (500 MHz, Chloroform-*d*) δ 8.56 (ddt, *J* = 19.5, 4.9, 1.2 Hz, 2H), 7.67 (qd, *J* = 7.9, 1.8 Hz, 2H), 7.29–7.16 (m, 2H), 7.24 (d, 1H), 7.03 (s, 2H), 5.25 (p, *J* = 6.8 Hz, 1H), 5.01–4.64 (m, 4H), 4.02 (dt, *J* = 11.4, 6.5 Hz, 6H), 1.79 (ddt, *J* = 36.0, 14.9, 6.7 Hz, 6H), 1.53 (d, *J* = 6.7 Hz, 3H), 1.52–1.44 (m, 6H), 1.32 (dd, *J* = 13.8, 6.0 Hz, 48H), 0.90 (t, *J* = 6.8 Hz, 9H) (Figure S12); ^13^C NMR (126 MHz, CDCl_3_) δ 173.86, 166.15, 156.68, 155.66, 153.08, 149.91, 141.24, 136.90, 128.95, 122.75, 122.45, 122.24, 121.54, 105.73, 73.50, 69.36, 52.73, 51.28, 46.29, 31.96, 31.94, 30.33, 29.77, 29.72, 29.72, 29.68, 29.66, 29.61, 29.43, 29.41, 29.38, 26.10, 22.71, 19.25, 14.13 (Figure S13); ESI-MS: calculated for C_58_H_95_N_4_O_5_ [M + H]+ 927.73 found 927.35; anal. calculated for C_58_H_95_N_4_O_5_: C, 75.12; H, 10.22; N, 6.04. Found: C, 75.21; H,10.34; N, 6.01.

### 3.5. Preparation of Supramolecular Gels

Gelator **1** (80 mg, 172 mmol) with and without Zn(OTf)_2_ were dissolved in 2 mL of ACN. The reaction mixtures were maintained at a constant reaction temperature of 25 °C for a set period of time to allow gel formation.

### 3.6. SEM Observation

FE-SEM (Tescan S8000 field emission SEM) was used to obtain images of the freeze-dried gel samples using an accelerating voltage 10–15 kV and an emission current of 10 mA. The gel samples were transferred into liquid N_2_ for 10 min and samples were then frozen at −40 °C overnight and freeze dried by vacuum at 0.1 Pa to yield the dry xerogels. (We used this instrument in National Research Facilities & Equipment Center: 2019R1A6C1010042).

### 3.7. Rheological Properties

The supramolecular gels were loaded onto the rheometer plate according the standard. Rheological properties were carried out by using AR-2000ex (TA Instruments Ltd.). The 20 mm diameter parallel plate was used. The setup of the gap between gel and plate was 1.0 mm and experiments were conducted at 25 °C. Strain sweep tests were performed with increasing amplitude oscillation from 0% to 200% apparent strain on shear. Frequency sweeps were performed from 0.6283 to 628.3 rad s^−1^.

## 4. Conclusions

We have demonstrated that two driving forces are responsible for the formation of bispicolylamine-based supramolecular gels, depending on the presence of Zn^2+^. Generally, hydrogen-bonding and π–π stacking were the main driving forces in the gel formation in the absence and presence of Zn^2+^, respectively. We also confirmed that a complex was formed between the gelator **1** and Zn^2+^. With 0.5 equiv. of Zn^2+^ in acetonitrile solvent, the gelator **1 c**omplexed with Zn^2+^ in 1:1. The uncomplexed **1** and [**1** + Zn + ACN]^2+^ complex coexisted, and the mechanical properties of the gel depreciated. At over 0.5 equiv., the coordination structure of the metal complex was replaced with anion (OTf) instead of acetonitrile. However, at >1 equiv., the gel solubilized completely as [**1** + Zn + OTf]^+^ formed. Here, we offer an advantageous process of making supramolecular gel in which the desired molecular properties could be achieved by altering the molecular structure during fabrication. By controlling the driving force, the network structure could be formed or dissociated. Therefore, we anticipate that this concept would provide useful information to overcome the disadvantages of degradability in material development.

## Supplementary Materials

Supplementary Materials can be found at https://www.mdpi.com/1422-0067/21/13/4617/s1.

## References

[1] Ye L., Zhang Y., Wang Q., Zhou X., Yang B., Ji F., Dong D., Gao L., Cui Y., Yao F. (2016). Physical Cross-Linking Starch-Based Zwitterionic Hydrogel Exhibiting Excellent Biocompatibility, Protein Resistance, and Biodegradability. ACS Appl. Mater. Interfaces.

[2] Ruiz-Olles J., Slavik P., Whitelaw N.K., Smith D.K. (2019). Self-Assembled Gels Formed in Deep Eutectic Solvents: Supramolecular Eutectogels with High Ionic Conductivity. Angew. Chem..

[3] Hamilton T.D., Bucar D.-K., Baltrusaitis J., Flanagan D.R., Li Y., Ghorai S., Tivanski A.V., MacGillivray L.R. (2011). Thixotropic Hydrogel Derived from a Product of an Organic Solid-State Synthesis: Properties and Densities of Metal-Organic Nanoparticles. J. Am. Chem. Soc..

[4] Aggeli A., Bell M., Boden N., Keen J.N., Knowles P.F., McLeish T.C., Pitkeathly M., Radford S.E. (1997). Responsive gels formed by the spontaneous self-assembly of peptides into polymeric beta-sheet tapes. Nature.

[5] Li B., He T., Fan Y., Yuan X., Qiu H., Yin S. (2019). Recent developments in the construction of metallacycle/metallacage-cored supramolecular polymers via hierarchical self-assembly. Chem. Commun..

[6] Li B., He T., Shen X., Tang D., Yin S. (2019). Fluorescent supramolecular polymers with aggregation induced emission properties. Polym. Chem..

[7] Yan X., Wang F., Zheng B., Huang F. (2012). Stimuli-responsive supramolecular polymeric materials. Chem. Soc. Rev..

[8] Stuart M.A.C., Huck W.T.S., Genzer J., Müller M., Ober C., Stamm M., Sukhorukov G.B., Szleifer I., Tsukruk V.V., Urban M. (2010). Emerging applications of stimuli-responsive polymer materials. Nat. Mater..

[9] Yu G., Zhao X., Zhou J., Mao Z., Huang X., Wang Z., Hua B., Liu Y., Zhang F., He Z. (2018). Supramolecular Polymer-Based Nanomedicine: High Therapeutic Performance and Negligible Long-Term Immunotoxicity. J. Am. Chem. Soc..

[10] Yan X., Liu Z., Zhang Q., Lopez J., Wang H., Wu H.-C., Niu S., Yan H., Wang S., Lei T. (2018). Quadruple H-Bonding Cross-Linked Supramolecular Polymeric Materials as Substrates for Stretchable, Antitearing, and Self-Healable Thin Film Electrodes. J. Am. Chem. Soc..

[11] Ma C., Lu W., Yang X., He J., Le X., Wang L., Zhang J., Serpe M.J., Huang Y., Chen T. (2018). Bioinspired Anisotropic Hydrogel Actuators with On–Off Switchable and Color-Tunable Fluorescence Behaviors. Adv. Funct. Mater..

[12] Cui Y.-H., Deng R., Li Z., Du X.-S., Jia Q., Wang X.-H., Wang C.-Y., Meguellati K., Yang Y.-W. (2019). Pillar [5]arene pseudo[1]rotaxane-based redox-responsive supramolecular vesicles for controlled drug release. Mater. Chem. Front..

[13] Yin Z., Song G., Jiao Y., Zheng P., Xu J.-F., Zhang X. (2019). Dissipative Supramolecular Polymerization Powered by Light. CCS Chem..

[14] Albrecht M. (2001). “Let’s Twist Again” Double-Stranded, Triple-Stranded, and Circular Helicates. Chem. Rev..

[15] Wan Q., To W.-P., Yang C., Che C.-M. (2018). The Metal–Metal-to-Ligand Charge Transfer Excited State and Supramolecular Polymerization of Luminescent Pincer PdII–Isocyanide Complexes. Angew. Chem..

[16] Tsai J.L.-L., Zou T., Liu J., Chen T., Chan A.O.-Y., Yang C., Lok C.-N., Che C.-M. (2015). Luminescent platinum(ii) complexes with self-assembly and anti-cancer properties: Hydrogel, pH dependent emission color and sustained-release properties under physiological conditions. Chem. Sci..

[17] Mamiya F., Ousaka N., Yashima E. (2015). Remote Control of the Planar Chirality in Peptide-Bound Metallomacrocycles and Dynamic-to-Static Planar Chirality Control Triggered by Solvent-Induced 310-to-α-Helix Transitions. Angew. Chem..

[18] Ousaka N., Shimizu K., Suzuki Y., Iwata T., Itakura M., Taura D., Iida H., Furusho Y., Mori T., Yashima E. (2018). Spiroborate-Based Double-Stranded Helicates: Meso-to-Racemo Isomerization and Ion-Triggered Springlike Motion of the Racemo-Helicate. J. Am. Chem. Soc..

[19] Robinson M.E., Nazemi A., Lunn D.J., Hayward D.W., Boott C.E., Hsiao M.-S., Harniman R.L., Davis S.A., Whittell G.R., Richardson R.M. (2017). Dimensional Control and Morphological Transformations of Supramolecular Polymeric Nanofibers Based on Cofacially-Stacked Planar Amphiphilic Platinum (II) Complexes. ACS Nano.

[20] Park H., Kim K.Y., Jung S.H., Choi Y., Sato H., Jung J.H. (2018). Different Origins of Strain-Induced Chirality Inversion of Co^2+^-Triggered Supramolecular Peptide Polymers. Chem. Mater..

[21] Nakamura T., Kimura H., Okuhara T., Yamamura M., Nabeshima T. (2016). A Hierarchical Self-Assembly System Built Up from Preorganized Tripodal Helical Metal Complexes. J. Am. Chem. Soc..

[22] Whittell G.R., Hager M.D., Schubert U.S., Manners I. (2011). Functional soft materials from metallopolymers and metallosupramolecular polymers. Nat. Mater..

[23] Winter A., Schubert U.S. (2016). Synthesis and characterization of metallo-supramolecular polymers. Chem. Soc. Rev..

[24] Higuchi M. (2014). Stimuli-responsive metallo-supramolecular polymer films: Design, synthesis and device fabrication. J. Mater. Chem. C.

[25] Yu L., Wang Z., Wu J., Tu S., Ding K. (2010). Directed Orthogonal Self-Assembly of Homochiral Coordination Polymers for Heterogeneous Enantioselective Hydrogenation. Angew. Chem..

[26] Langenstroer A., Dorca Y., Kartha K.K., Mayoral M.J., Stepanenko V., Fernández G., Sánchez L. (2018). Exploiting N—H···Cl Hydrogen Bonding Interactions in Cooperative Metallosupramolecular Polymerization. Macromol. Rapid Commun..

[27] Taura D., Hioki S., Tanabe J., Ousaka N., Yashima E. (2016). Cobalt (II)-Salen-Linked Complementary Double-Stranded Helical Catalysts for Asymmetric Nitro-Aldol Reaction. ACS Catal..

[28] Chen L.-J., Yang H.-B., Shionoya M. (2017). Chiral metallosupramolecular architectures. Chem. Soc. Rev..

[29] Chan M.H.-Y., Leung S.Y.-L., Yam V.W.-W. (2018). Controlling Self-Assembly Mechanisms through Rational Molecular Design in Oligo (p-phenyleneethynylene)-Containing Alkynylplatinum (II) 2,6-Bis (N-alkylbenzimidazol-2′-yl) pyridine Amphiphiles. J. Am. Chem. Soc..

[30] Leung S.Y.-L., Wong K.M.-C., Yam V.W.-W. (2016). Self-assembly of alkynylplatinum (II) terpyridine amphiphiles into nanostructures via steric control and metal-metal interactions. Proc. Natl. Acad. Sci. USA.

[31] Wong V.C.-H., Po C., Leung S.Y.-L., Chan A.K.-W., Yang S., Zhu B., Cui X., Yam V.W.-W. (2018). Formation of 1D Infinite Chains Directed by Metal-Metal and/or π-π Stacking Interactions of Water-Soluble Platinum (II) 2,6-Bis (benzimidazol-2’-yl) pyridine Double Complex Salts. J. Am. Chem. Soc..

[32] Wan Q., Xiao X.-S., To W.-P., Lu W., Chen Y., Low K.-H., Che C.-M. (2018). Counteranion- and Solvent-Mediated Chirality Transfer in the Supramolecular Polymerization of Luminescent Platinum (II) Complexes. Angew. Chem..

[33] Kim K.Y., Kim J., Moon C.J., Liu J., Lee S.S., Choi M.Y., Feng C., Jung J.H. (2019). Co-Assembled Supramolecular Nanostructure of Platinum (II) Complex through Helical Ribbon to Helical Tubes with Helical Inversion. Angew. Chem..

[34] Lee J.H., Jaworski J., Jung J.H. (2013). Luminescent metal-organic framework-functionalized graphene oxide nanocomposites and the reversible detection of high explosives. Nanoscale.

[35] O’Neil E.J., Smith B.D. (2006). Anion recognition using dimetallic coordination complexes. Coord. Chem. Rev..

[36] Lacoste R.G., Marttel A.E. (1964). New Multidentate Ligands. I. Coordinating Tendencies of Polyamines Contaimng α-Pyridyl Groups with Divalent Metal Ions. Inorg. Chem..

[37] Gultneh Y., Khan A.R., Blaise D., Chaudhry S., Ahvazi B., Marvey B.B., Butcher R.J. (1999). Syntheses and structures of and catalysis of hydrolysis by Zn (II) complexes of chelating pyridyl donor ligands. J. Inorg. Biochem..

[38] Mokhir A., Krämer R., Wolf H. (2004). Zn^2+^-Dependent Peptide Nucleic Acids Probes. J. Am. Chem. Soc..

[39] Jiang P., Guo Z. (2004). Fluorescent detection of zinc in biological systems: Recent development on the design of chemosensors and biosensors. Coord. Chem. Rev..

[40] Walkup G.K., Burdette S.C., Lippard S.J., Tsien R.Y. (2000). A New Cell-Permeable Fluorescent Probe for Zn^2+^. J. Am. Chem. Soc..

[41] Lim N.C., Brückner C. (2004). DPA-substituted coumarins as chemosensors for zinc (ii): Modulation of the chemosensory characteristics by variation of the position of the chelate on the coumarin. Chem. Commun..

[42] Chang C.J., Nolan E.M., Jaworski J., Burdette S.C., Sheng M., Lippard S.J. (2004). Bright Fluorescent Chemosensor Platforms for Imaging Endogenous Pools of Neuronal Zinc. Chem. Biol..

[43] Ojida A., Mito-oka Y., Sada K., Hamachi I. (2004). Molecular Recognition and Fluorescence Sensing of Monophosphorylated Peptides in Aqueous Solution by Bis (zinc (II)−dipicolylamine)-Based Artificial Receptors. J. Am. Chem. Soc..

[44] Ojida A., Inoue M.-a., Mito-oka Y., Hamachi I. (2003). Cross-Linking Strategy for Molecular Recognition and Fluorescent Sensing of a Multi-phosphorylated Peptide in Aqueous Solution. J. Am. Chem. Soc..

[45] Lee D.H., Kim S.Y., Hong J.-I. (2004). A Fluorescent Pyrophosphate Sensor with High Selectivity over ATP in Water. Angew. Chem..

[46] Lee D.H., Im J.H., Son S.U., Chung Y.K., Hong J.-I. (2003). An Azophenol-based Chromogenic Pyrophosphate Sensor in Water. J. Am. Chem. Soc..

[47] Jiang H., O’Neil E.J., DiVittorio K.M., Smith B.D. (2005). Anion-Mediated Phase Transfer of Zinc (II)-Coordinated Tyrosine Derivatives. Org. Lett..

[48] Routasalo T., Helaja J., Kavakka J., Koskinen A.M.P. (2008). Development of Bis(2-picolyl)amine–Zinc Chelates for Imidazole Receptors. Eur. J. Org. Chem..

[49] Kim Y., Park B.K., Eom G.H., Kim S.H., Park H.M., Choi Y.S., Jang H.G., Kim C. (2011). Anion effects on construction of ZnII compounds with a chelating ligand bis (2-pyridylmethyl) amine and their catalytic activities. Inorg. Chim. Acta.

[50] Schmidt M., Goerls H., Plass W. (2016). Facile high-yield synthesis of unsymmetric end-off compartmental double Schiff-base ligands: Easy access to mononuclear precursor and unsymmetric dinuclear complexes. RSC Adv..

[51] Skalamera D., Sanders E., Vianello R., Marsavelski A., Pevec A., Turel I., Kirin S.I. (2016). Synthesis and characterization of ML and ML2 metal complexes with amino acid substituted bis (2-picolyl) amine ligands. Dalton Trans..

[52] Pantalon Juraj N., Muratović S., Perić B., Šijaković Vujičić N., Vianello R., Žilić D., Jagličić Z., Kirin S.I. (2020). Structural Variety of Isopropyl-bis (2-picolyl) amine Complexes with Zinc (II) and Copper (II). Cryst. Growth Des..

[53] Qian H., Aprahamian I. (2015). An emissive and pH switchable hydrazone-based hydrogel. Chem. Commun..

[54] Appel E.A., del Barrio J., Loh X.J., Scherman O.A. (2012). Supramolecular polymeric hydrogels. Chem. Soc. Rev..

[55] Zhang M., Xu D., Yan X., Chen J., Dong S., Zheng B., Huang F. (2012). Self-Healing Supramolecular Gels Formed by Crown Ether Based Host–Guest Interactions. Angew. Chem..

[56] Yan X., Xu D., Chi X., Chen J., Dong S., Ding X., Yu Y., Huang F. (2012). A Multiresponsive, Shape-Persistent, and Elastic Supramolecular Polymer Network Gel Constructed by Orthogonal Self-Assembly. Adv. Mater..

[57] Anokhin D.V., Lejnieks J., Mourran A., Zhu X., Keul H., Moeller M., Konovalov O., Erina N., Ivanov D.A. (2012). Interplay between H-Bonding and Alkyl-Chain Ordering in Self-Assembly of Monodendritic L-Alanine Derivatives. ChemPhysChem.

[58] Zhang S., Sun H.-J., Hughes A.D., Draghici B., Lejnieks J., Leowanawat P., Bertin A., Otero De Leon L., Kulikov O.V., Chen Y. (2014). “Single-Single” Amphiphilic Janus Dendrimers Self-Assemble into Uniform Dendrimersomes with Predictable Size. ACS Nano.

