# Supplementary material for: Bispicolyamine-Based Supramolecular Polymeric Gels Induced by Distinct Different Driving Forces with and Without Zn2+
Source: Int J Mol Sci. 2020 Jun 29;21(13):4617. doi: 10.3390/ijms21134617 (PMC7369882; doi:10.3390/ijms21134617)
Supplement: Supplementary file 1 [file ijms-21-04617-s001.pdf]

Supplementary Materials

Bispicolyamine-based supramolecular polymeric gels induced by distinct different driving forces with and without Zn^{2+}

Jaehyeon Park[1,ǂ], Ka Young Kim[1,ǂ], Seok Gyu Kang[1], Shim Sung Lee[1], Ji Ha Lee[2,*] and Jong Hwa Jung[1,*]

1 Department of Chemistry and Research Institute of Natural Sciences, Gyeongsang National University, Jinju; 52828, Korea; parkjae@gnu.ac.kr (J.P), rk5321@gnu.ac.kr (G.Y.K) dsa61milk@naver.com (S.G.K) sslee@gnu.ac.kr (S.S.L), jonghwa@gnu.ac.kr (J.H.J)

2 Chemical Engineering Program, Graduate School of Advanced Science and Engineering, Hiroshima University, 1-4-1 Kagamiyama, Higashi-Hiroshima, Hiroshima 739-8527, Japan; leejiha@hiroshima-u.ac.jp (J.H.L)

ǂ These authors contributed equally in this work

* Correspondence: leejiha@hiroshima-u.ac.jp (J.H.L); jonghwa@gnu.ac.kr; Tel.: +82-55-772-1488 (J.H.J)

Table S1. Gelation Test of **1** without and with Zn^{2+} (0.5equiv.).

Solvent	State	
	Without Zn^{2+}	With Zn^{2+}
Toluene	S	S
DCM	S	S
Chloroform	S	S
THF	S	S
Acetone	S	S
MCH	S	S
Ethyl acetate	S	S
Ethyl ether	S	S
DMSO	PG	S
EtOH	PG	S
ACN	G	S
MeOH	G	S
n-hexane	G	S
H_2O	I	I

S: Solution, PG: Partial gel, G: Gel, I: Insoluble.

Table S2. ESI-MS results of **1** in several equivalents of Zn^{2+}.

	m/z	Equivalent of Zn^{2+}						
		0.3	0.5	0.6	0.7	0.8	0.9	1.1
[**1**+Zn+ACN]$^{2+}$	515.84	100%	51%	40%	10%	4%	-	-
[**1**+ZnOTf]$^{+}$	1139.60	0%	49%	60%	90%	96%	100%	100%

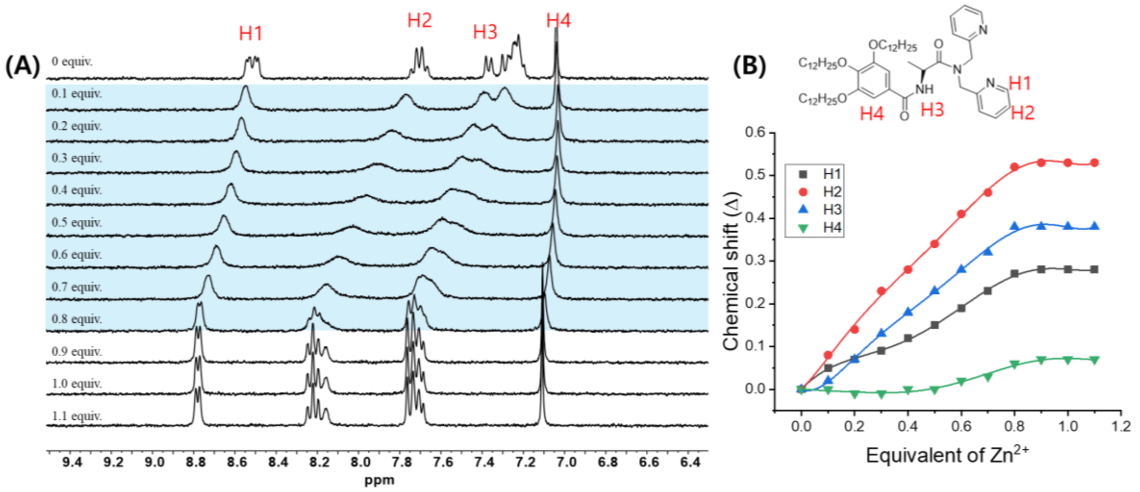

Figure S1. (A) ^{1}H NMR spectra of **1** (2.1 μM) with different concentration of Zn^{2+} (0 – 1.1 equivalent) in ACN-d_3 at 50 °C. (B) Plot of chemical shift changes of **1** against various concentrations of Zn^{2+}.

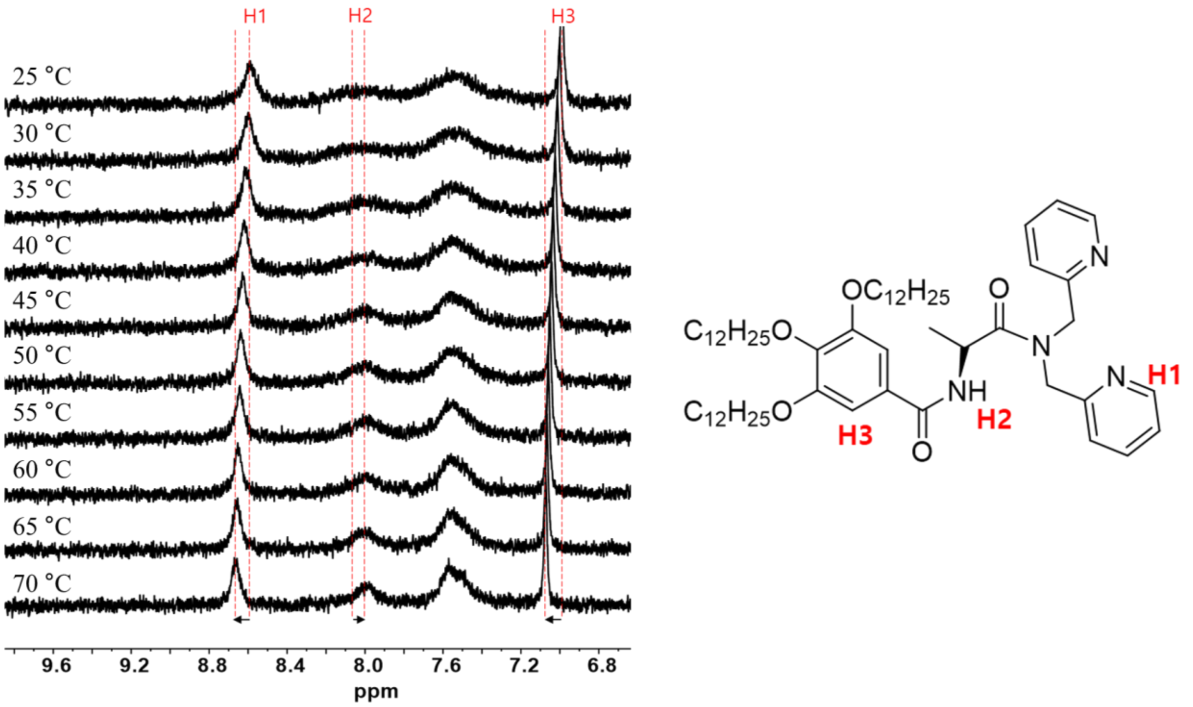

Figure S2. Temperature-dependent NMR spectra of **1** (2.1 μM) in ACN-*d3*.

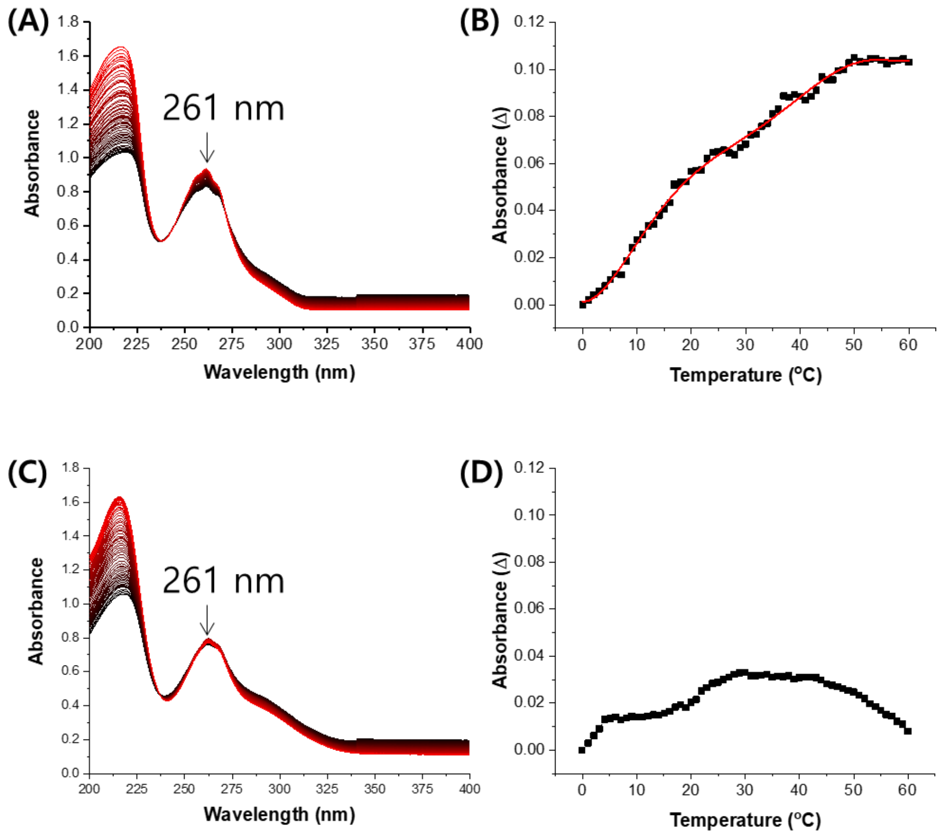

Figure S3. (A) Temperature-dependent UV-vis spectra of **1** (0.05 mM) in ACN. (B) Plot of absorbance change of **1** as a function of temperature changes at 261 nm. (C) Temperature-dependent UV-vis spectra of **1** (0.05 mM) with Zn^{2+} (0.025 mM) in ACN. (D) Plot of absorbance change of **1** with Zn^{2+} as a function of temperature changes at 261 nm.

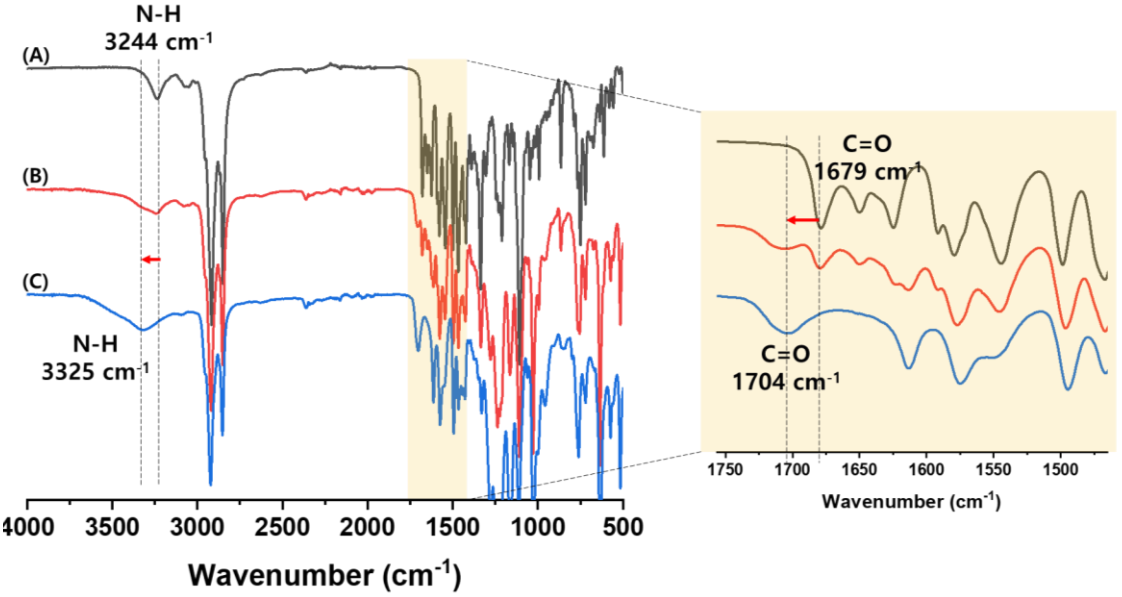

Figure S4. IR spectra of **1** with (A) 0 equiv. (B) 0.5 equiv. and (C) 1.0 equiv. of Zn^{2+}.

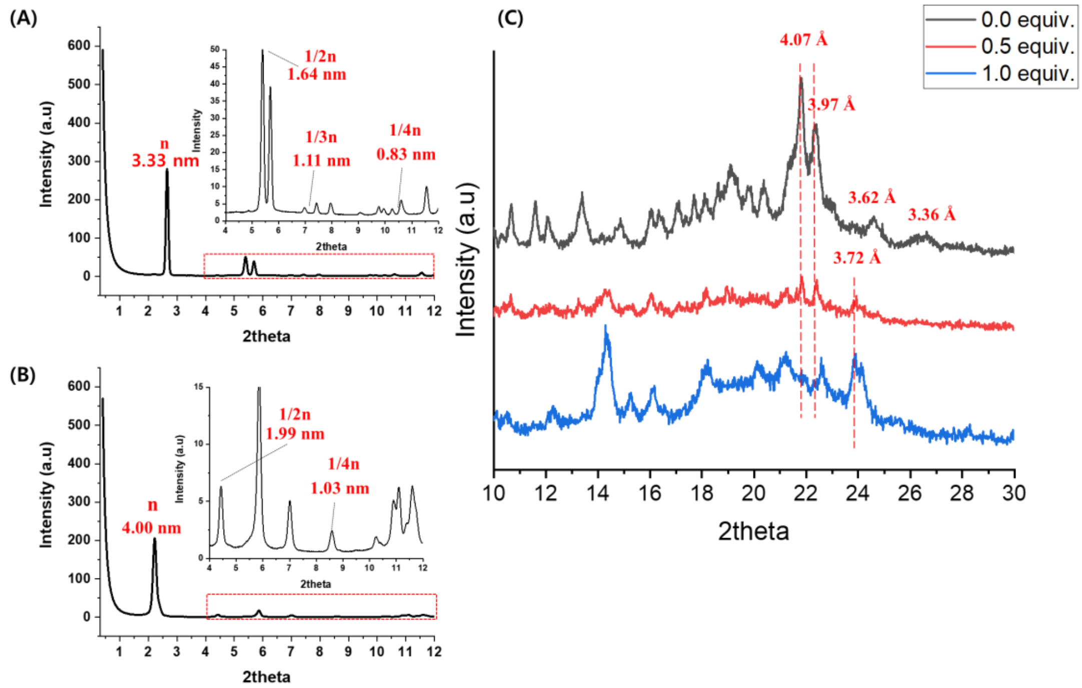

Figure S5. Small-angle X-ray scattering patterns of **1** (A) without and (B) with Zn^{2+}. (C) Powder XRD patterns of **1** with various equivalent of Zn^{2+}.

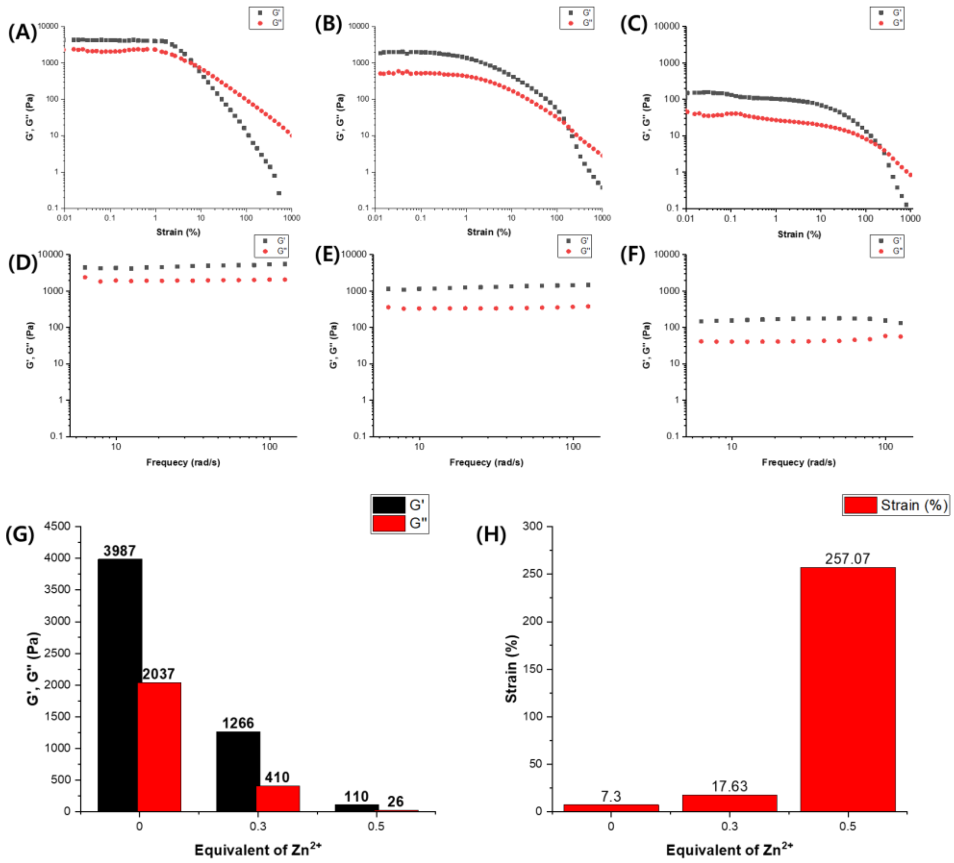

Figure S6. Strain sweep at 0.1 % - 1000 % (frequency = 0.6283 $rads^{-1}$) of **1** with (A) 0 equiv. (B) 0.3 equiv. and (C) 0.5 equiv. of Zn^{2+}. Frequency sweep of **1** with (D) 0 equiv. (E) 0.3 equiv. and (F) 0.5 equiv. of Zn^{2+} at a strain of 0.1 %. (G) Bar graph for G′ and G″ values at γ = 0.1% in (A)-(C). (H) Graph of γ values at G″/G′=1 in (A)-(C).

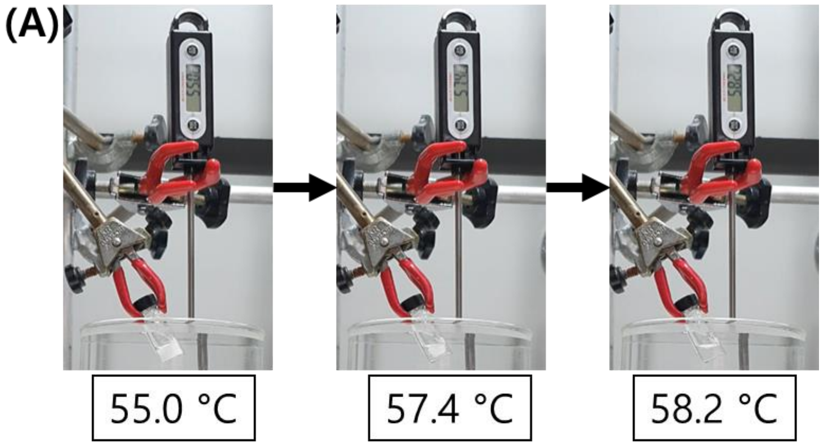

Figure S7. Photographs of **1** (A) without and (B) with Zn^{2+} (0.5 equiv.) after heating in the water bath.

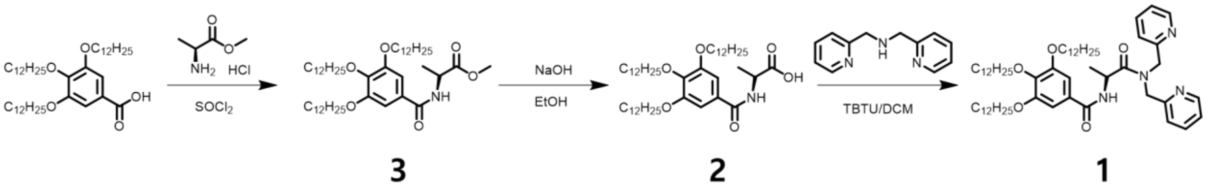

Scheme S1. Schematic of synthetic methods for **1**.

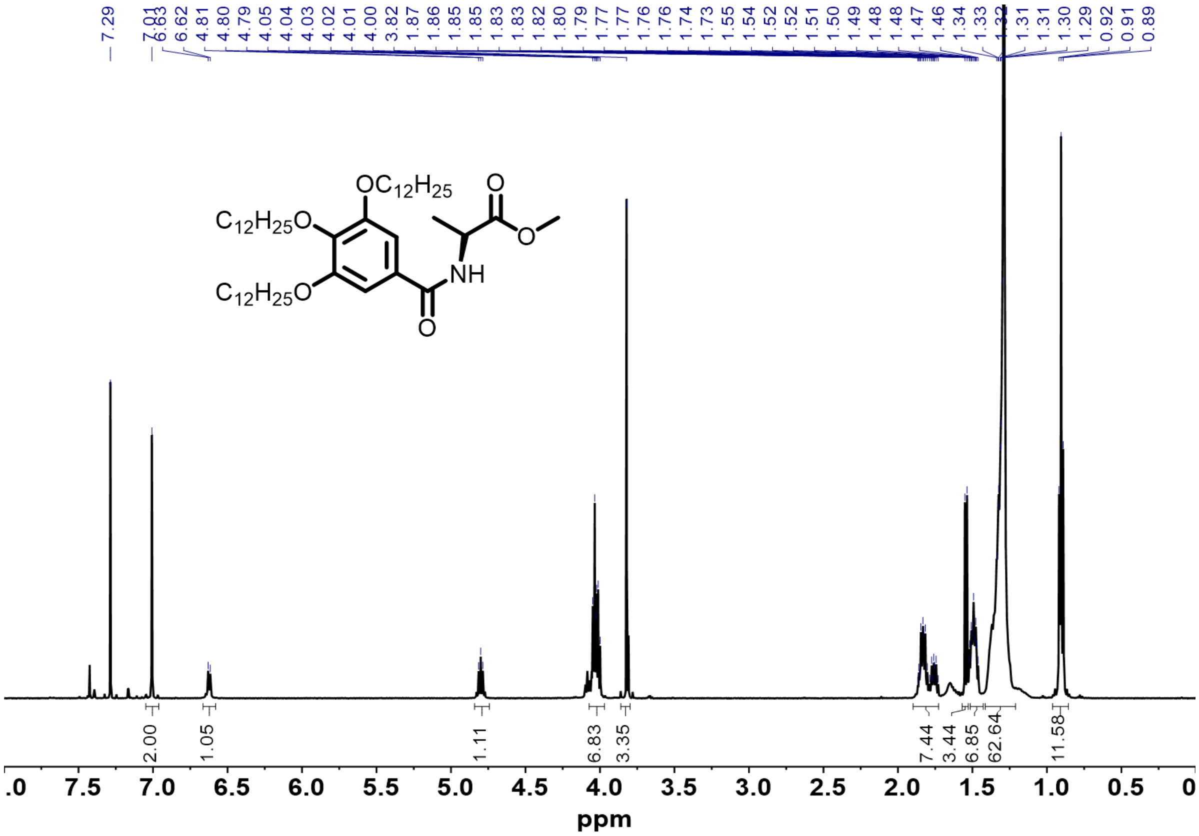

Figure S8. ^{1}H NMR spectrum of **3** in $CDCl_3$.

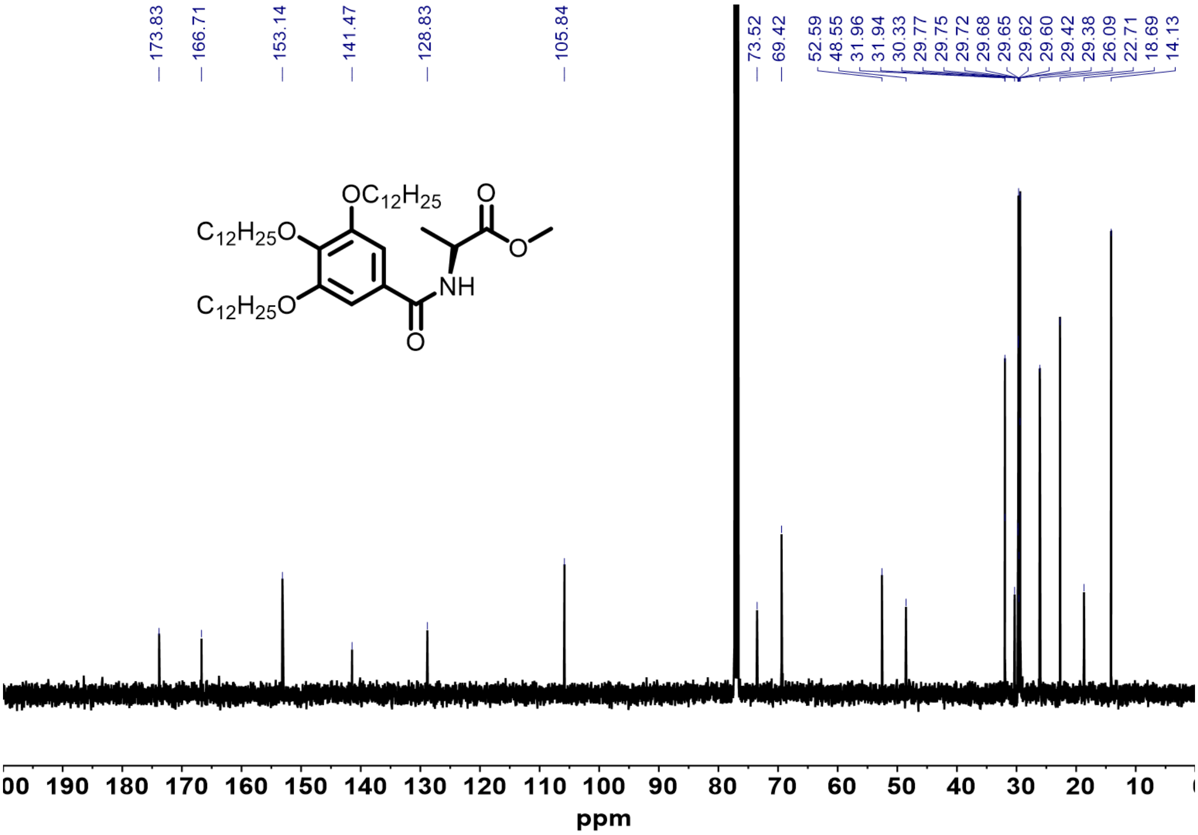

Figure S9. ^{13}C NMR spectrum of **3** in $CDCl_3$.

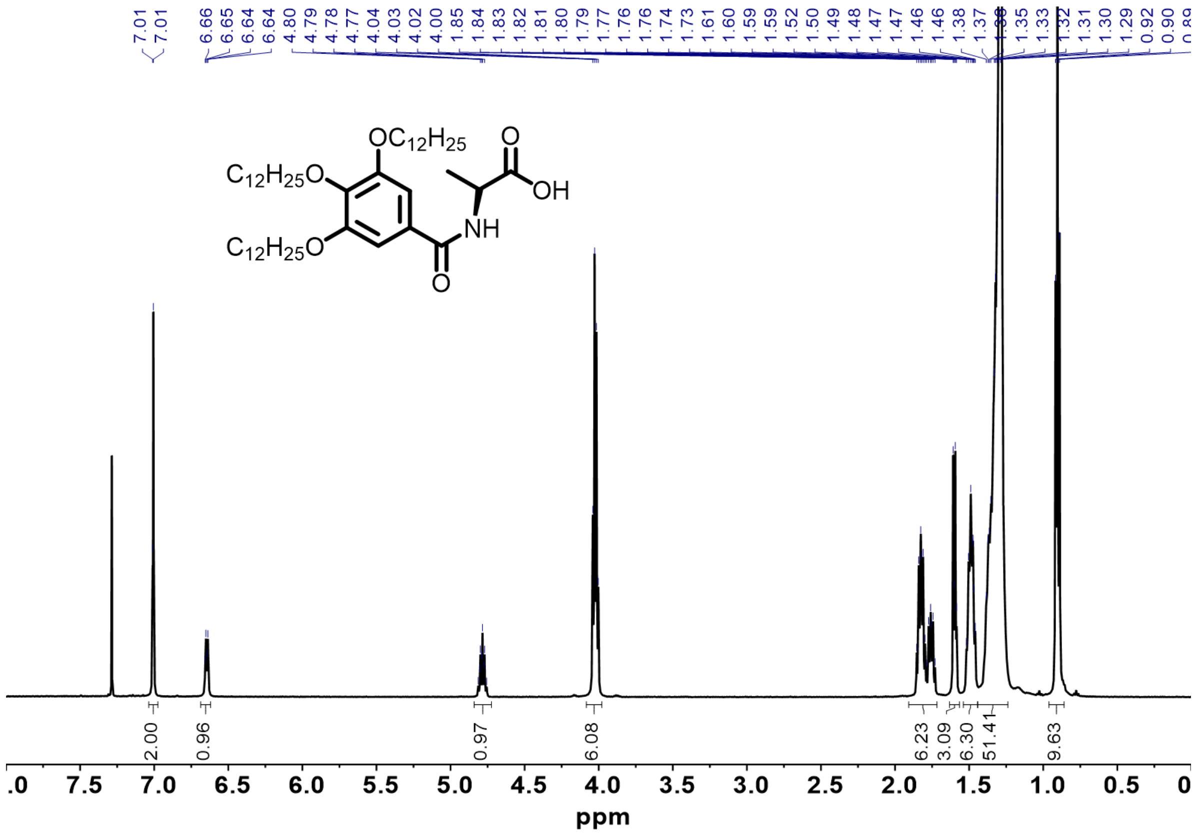

Figure S10. ^{1}H NMR spectrum of **2** in $CDCl_3$.

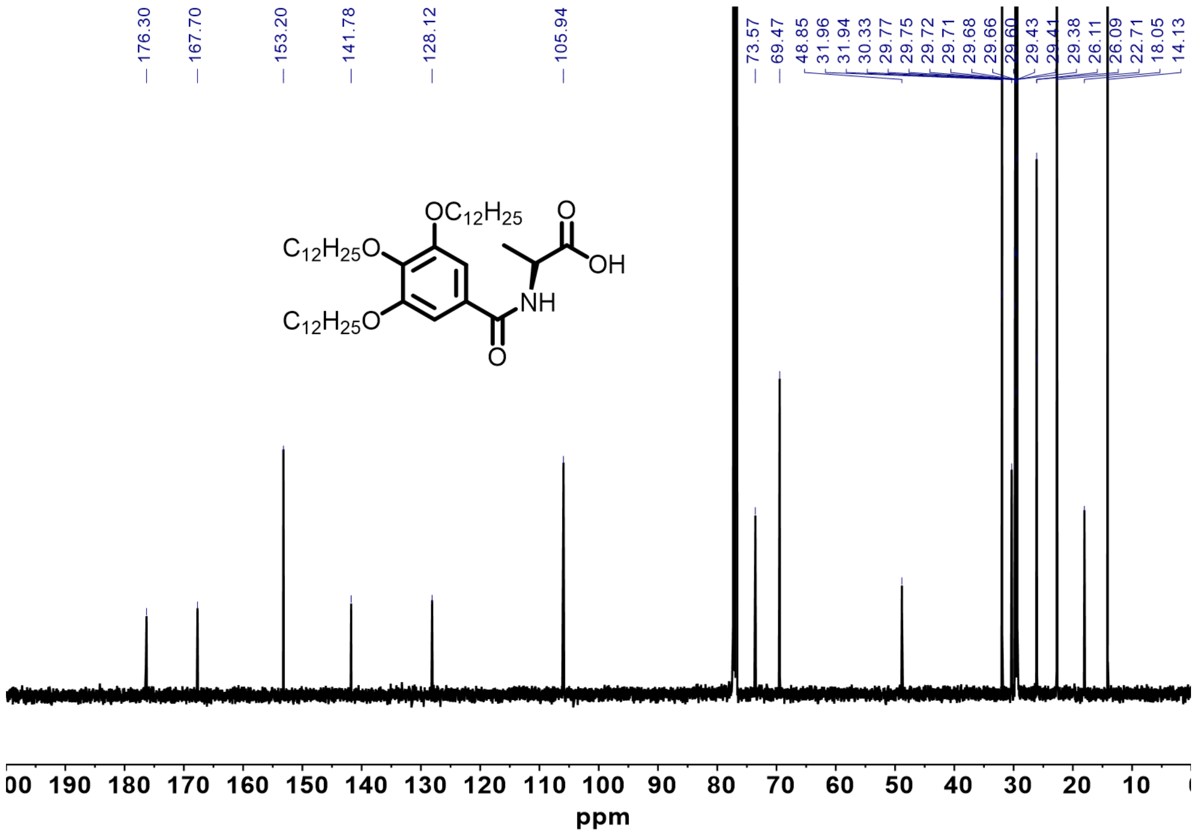

Figure S11. ^{13}C NMR spectrum of **2** in $CDCl_3$.

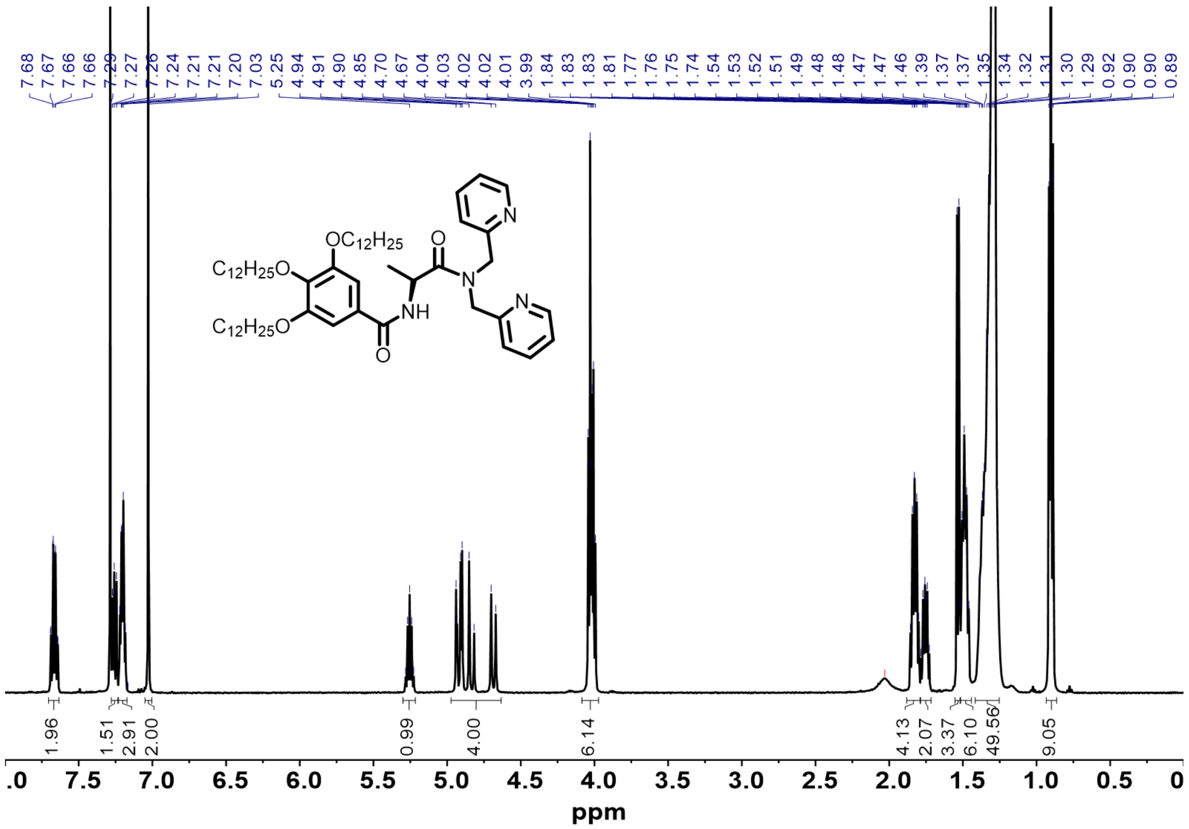

Figure S12. ^{1}H NMR spectrum of **1** in $CDCl_3$.

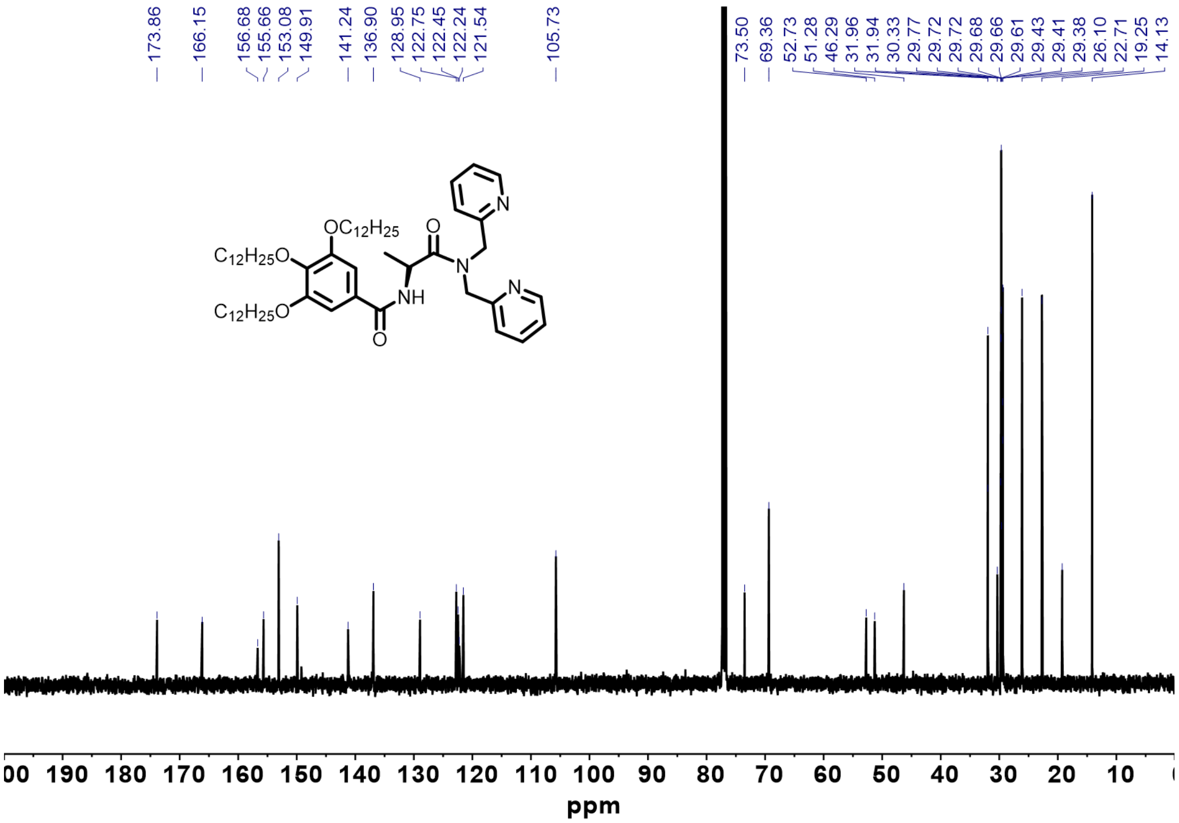

Figure S13. ^{13}C NMR spectrum of **1** in $CDCl_3$.
